# Supplementary material for: Evaluation of a Low-threshold Exercise And Protein supplementation intervention for Women (LEAP-W) experiencing homelessness and addiction: Protocol for a single-arm mixed methods feasibility study
Source: PLoS One. 2025 Feb 6;20(2):e0300412. doi: 10.1371/journal.pone.0300412 (PMC11801605; doi:10.1371/journal.pone.0300412)
Supplement: S1 File — (DOCX) [file pone.0300412.s001.docx]

**S1: Data Collection Form LEAP-W**

| **Unique Identifier No:** |
| --- |
| **CONSENT** |
| **Date** |
| **GP** |
| **Ethnicity** |
| **Age** |
| **Sex** |
| **Living arrangement** |
| **Living environment** |
| **Education (1°, 2°, 3°)** |
| **Employment status** |
| **Marital status** |
| **H/o incarceration** |
| **H/o addiction** |
| **Self-reported health conditions** |
| **Women’s health** (no of children, pre-post menopause) |
| **Clinical Frailty Scale** |
| **Pain-Numerical Pain Rating Scale*/location/>3 months*** |
| **Mini Nutritional Assessment score** |
| **SHARE-Frailty Instrument score** |
| **Short-Form 12 score** |
| **Limb circumference** |
| **BP/HR** |
| **Dynamometry** |
| **Chair Stand Test** |
| **10mWT** |
| **2minWT** |
| **Single Leg Stance** |
