# Supplementary material for: Evaluation of a Low-threshold Exercise And Protein supplementation intervention for Women (LEAP-W) experiencing homelessness and addiction: Protocol for a single-arm mixed methods feasibility study
Source: PLoS One. 2025 Feb 6;20(2):e0300412. doi: 10.1371/journal.pone.0300412 (PMC11801605; doi:10.1371/journal.pone.0300412)
Supplement: S2 File — (DOCX) [file pone.0300412.s002.docx]

**S2: Interview Schedule**

Intro: “We’re here to talk about the LEAP-W exercise programme ...

1. Can you tell me why did you agree to take part in the first place?

*Prompts: to change life around; to improve my health; to get back into exercise; to reduce substance use; was encouraged to; attended before?*

1. What did you enjoy about the programme?

*Prompts: exercise classes/Park Walk/music/the group dynamic/exercise options/women’s health focus*

1. What benefits, if any, did you get from the programme?

*Prompts: Short term (immediately after the class)/Long term (from beginning to end)*

1. Did your substance use change over the course of the programme? In what way?

*Prompts: reduced/increased use/how much*

1. Has attending the programme changed your views of/future plans to take part in physical activity/exercise activities?

*Prompts: In what way? Would you do the Park Walk on a regular basis now? With others/friends?*

1. Can you tell me what, if anything, made it hard for you to attend?
2. Is there anything that you didn’t like about the exercise programme?
3. Is there anything that you think of which would have made it better/easier for you to attend? What would you change?
4. Is there anything else that you would like to add?

Thank you.
