# Supplementary material for: Evaluation of a Low-threshold Exercise And Protein supplementation intervention for Women (LEAP-W) experiencing homelessness and addiction: Protocol for a single-arm mixed methods feasibility study
Source: PLoS One. 2025 Feb 6;20(2):e0300412. doi: 10.1371/journal.pone.0300412 (PMC11801605; doi:10.1371/journal.pone.0300412)
Supplement: S3 File — (DOC) [file pone.0300412.s003.doc]

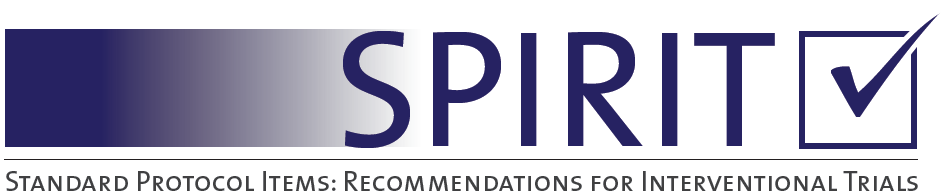


**S3:** SPIRIT 2013 Checklist: Recommended items to address in a clinical trial protocol and related documents*

| Section/item | ItemNo | Description |
| --- | --- | --- |
| **Administrative information** | | |
| Title | 1 | Evaluation of a **L**ow-threshold **E**xercise **A**nd **P**rotein supplementation intervention for **W**omen (**LEAP-W**) experiencing homelessness and addiction: Protocol for a single-arm mixed methods feasibility study. |
| Trial registration | 2a | Clinical Trials.gov number: NCT05700305 |
| Protocol version | 3 | 14.03.24 Version 1 |
| Funding | 4 | This study has been funded by The University of Dublin, Trinity College Dublin and the Irish Research Council. |
| Roles and responsibilities | 5a | Authors: Fiona Kennedy ^1^, Deirdre Murray, ^2^, Clíona Ní Cheallaigh ^3,4^, Roman Romero-Ortuno ^5,6,7^, Julie Broderick ^1^.  Affiliations: ^1^Discipline of Physiotherapy, School of Medicine, Trinity College Dublin, Dublin, D08W9RT, Ireland  ^2^Academic Unit of Neurology, Trinity Biomedical Sciences Institute, Trinity College Dublin, Dublin, D02 R590, Ireland.  ^3^ St James's Hospital, Dublin, D08W9RT, Ireland.  ^4^Department of Clinical Medicine, School of Medicine, Trinity College Dublin, Dublin, D08W9RT, Ireland.  ^5^Discipline of Medical Gerontology, School of Medicine, Trinity College Dublin, Dublin, D08 NHY1, Ireland.  ^6^Mercer’s Institute for Successful Ageing, St James’s Hospital, Dublin, D08 NHY1, Ireland.  ^7^Global Brain Institute, Trinity College Dublin, D02 R590, Ireland.  Roles of protocol contributors: FK and JB contributed to the research concept and design, visualisation and drafting the manuscript. RR, CnC and DM contributed to visualisation, validation and drafting of the manuscript. All authors read and approved the final manuscript. |
|  | 5b | Trial sponsor: Trinity College Dublin, The University of Dublin, College Green, Dublin 2, Ireland. |
|  | 5c | Role of study sponsor and funders, if any, in study design; collection, management, analysis, and interpretation of data; writing of the report; and the decision to submit the report for publication, including whether they will have ultimate authority over any of these activities:  Study funders will have no role in the study design; collection, management, analysis, and interpretation of data; writing of the report; and the decision to submit the report for publication, including whether they will have ultimate authority over any of these activities. |
|  | 5d | Composition, roles, and responsibilities of the coordinating centre, steering committee, endpoint adjudication committee, data management team, and other individuals or groups overseeing the trial, if applicable (see Item 21a for data monitoring committee):  This study does not have a formal Data Monitoring Committee. It is being undertaken by Fiona Kennedy as part of her PhD and is supervised by the Principal Investigator, Dr. Julie Broderick and co-supervisor Dr. Deirdre Murray. Prof Roman Romero-Ortuno and Prof Clíona Ní Cheallaigh are study collaborators and will collaborate and advise on the data interpretation and reporting. |

| Introduction |  |  |
| --- | --- | --- |
| Background and rationale | 6a | Description of research question and justification for undertaking the trial, including summary of relevant studies (published and unpublished) examining benefits and harms for each intervention:  Frailty is a complex multi-dimensional state of increased vulnerability to adverse health outcomes and is usually associated with older age but there is growing evidence of accelerated ageing and frailty in non-geriatric at-risk populations, including those experiencing socio-economic deprivation and extreme social exclusion, such as people experiencing homelessness. Addiction, as a coping mechanism for prior trauma, is common among people who are homeless and can have a gendered dimension. Women experiencing homelessness and addiction have unique needs which require a gendered approach.  Targeted interventions to manage frailty in older adults are recommended and exercise with nutritional supplementation has proven effectiveness in combatting and mitigating frailty. Previous linked research from the LEAP I trial demonstrated high retention (83% female v 42% males) with excellent engagement and feedback from its female participants, yet caution was observed around engaging in a mixed exercised programme with men. Women have traditionally been underserved in research and policy, yet multiple sources cite women as the fastest growing homeless demographic.  No study has examined how targeted women-only exercise programmes with nutritional supplementation work in practice in this population.  The aim of this study is to explore the effectiveness of an exercise intervention to target the known physical functioning deficits and frailty which this population experiences. |
|  | 6b | Explanation for choice of comparators; This is a pre-post intervention study with no comparator group. |
| Objectives | 7 | Specific objectives or hypotheses:  1. To evaluate recruitment, retention and adherence rates, and any adverse effects of the intervention.  2. To evaluate pre- and post-intervention levels of physical, nutritional and frailty status, pain and general health status.  3. To evaluate programme feedback and gain deeper insights into participants perspectives and experiences of the exercise intervention. |
| Trial design | 8 | Description of trial design including type of trial (eg, parallel group, crossover, factorial, single group), allocation ratio, and framework (eg, superiority, equivalence, noninferiority, exploratory):  This is a single-arm quantitatively driven, sequential, mixed methods feasibility study involving a single group of women experiencing homelessness and addiction. It has two parts; 1) a pre-post intervention study to evaluate the impact of the intervention and 2) a qualitative study to explore perspectives of the value of the intervention. |
| Methods: Participants, interventions, and outcomes | | |
| Study setting | 9 | Description of study settings (eg, community clinic, academic hospital) and list of countries where data will be collected. Reference to where list of study sites can be obtained:  This is a single site study and will take place in a day-service centre (Jane’s Place) in Dublin city centre, Ireland, which provides services for women who are homeless and/or in addiction. |
| Eligibility criteria | 10 | Inclusion and exclusion criteria for participants:  Inclusion criteria:   - Adult women (18-65 years) accessing services in Jane’s Place who consent to participation.   Exclusion criteria:   - Any person not meeting the age eligibility criteria. - Any person identifying as a male. - Any person lacking capacity to give consent. - Any person with major physical/medical or cognitive challenges which would preclude ability to safely complete the assessment or - Any person with insufficient English language ability to give fully informed consent.   Individuals who will perform the interventions: The lead research physiotherapist and a physiotherapy research assistant will conduct the intervention. |
| Interventions | 11a | Interventions for each group with sufficient detail to allow replication, including how and when they will be administered:  The intervention will involve a 10-week low threshold exercise intervention (two exercise classes with protein supplementation and an outdoor ‘Park Walk’). Using a trauma-informed approach flexibly arranged group or one-to one sessions will be delivered based on participant preference. Exercise programming strategies and variables are based on our earlier work (LEAP I and LEAP II trials) and also informed by prior stakeholder involvement from people with lived experience. The exercise classes will be multi-modal, with a primary focus on strength and based on core set of resistance exercises (Table 3). Aerobic, balance and flexibility work will be integrated into the class and the exercises will be individualised based on initial assessment results and presentation of participants. Using a gender-based perspective, the following considerations and adaptions will be built into the class; (i) core-stability exercises to target pelvic floor and/or abdominal muscle weakness, (ii) bone building exercises to target peri-and postmenopausal bone loss and (iii) age-associated muscle mass loss. Music, dance and fun orientated physical activity games, an important feature of aforementioned linked studies, will be incorporated to optimize enjoyment and self-esteem. Borg’s Rate of Perceived Exertion Scale will be used to monitor effort and scale the intensity of the workout. To promote post-exercise muscle protein synthesis, a nutritional supplement (200ml pre-prepared ‘protein shake’, Fresubin) which consists of 20g of protein will be offered immediately post exercise. The ‘Park Walk’ will focus on the aerobic component of the intervention, using green open space to maximise physical and mental health outcomes. It will be a flexibly arranged 20–30-minute self-paced group or one-to-one walk. To build sustainability beyond the ten-week programme brief health promoting and physical activity educational interventions will be included in the exercise class setting to empower people to engage in unsupervised exercise following the study. |
|  | 11b | Criteria for discontinuing or modifying allocated interventions for a given trial participant (eg, drug dose change in response to harms, participant request, or improving/worsening disease):  In the unlikely event of an adverse event (unfavourable experience which occurs during the exercise intervention) participants will cease exercising, be managed by the study team and where indicated medical attention will be sought. |
|  | 11c | Strategies to improve adherence to intervention protocols, and any procedures for monitoring adherence (eg, drug tablet return, laboratory tests):  As per normal working procedure in the centre, reminders are routinely sent to service users regarding upcoming events or individual appointments. Similarly, phone calls or text messages will be sent by staff and researchers to remind participants of upcoming classes/walking interventions. Adherence will be monitored via programme attendance. |
|  | 11d | Relevant concomitant care and interventions that are permitted or prohibited during the trial: N/A |
| Outcomes | 12 | Primary, secondary, method of aggregation (eg, median, proportion), and time point for each outcome:  Primary Outcome- The primary outcome is feasibility measured by recruitment numbers, retention rate, adherence to the exercise programme and protein supplementation, programme acceptability and adverse events (Protocol, Table 1).    Secondary Outcome-Secondary outcomes are physical function, frailty, pain, nutritional status and self-reported health status (Protocol, Table 2).  Outcomes will be measured pre (week 0) and post intervention (week 10). Nominal or ordinal variables will be reported as frequencies and percentages. Continuous variables will be summarised as mean and standard deviation if normally distributed and median and inter-quartile range if non-normally distributed. Data will be tested for normality using the Kolmogorov–Smirnov/Shapiro Wilk test and will be compared across timepoints using the general linear model procedure (normally distributed data) and the Friedman’s test (non-normally distributed data).  Qualitative data will be firstly coded and theme generation will be conducted following this using Braun and Clarke methodology. |
| Participant timeline | 13 | Time schedule of enrolment, interventions (including any run-ins and washouts), assessments, and visits for participants:  Participants will be recruited from February to April 2024 and the intervention will be of 10 weeks duration. To provide flexibility and maximum participation the 10 weeks commencement date will be staggered until the end of the recruitment period. Participants can attend up to 3 times/week for the 2 exercise classes and the once weekly Park Walk. |
| Sample size | 14 | Estimated number of participants needed to achieve study objectives and how it was determined, including clinical and statistical assumptions supporting any sample size calculations:  Sample sizes of at least 24 participants are recommended for feasibility studies and will be the minimal target for this study. |
| Recruitment | 15 | Strategies for achieving adequate participant enrolment to reach target sample size:  A prior stakeholder engagement session featuring education and co-design will be conducted to encourage recruitment and plain language information sheets will be distributed in the centre and adjacent centres. |
| **Methods: Assignment of interventions (for controlled trials)** | | |
| Allocation: This is a pre-post intervention study with no comparator group |  |  |
| Sequence generation | 16a | Method of generating the allocation sequence (eg, computer-generated random numbers), and list of any factors for stratification. To reduce predictability of a random sequence, details of any planned restriction (eg, blocking) should be provided in a separate document that is unavailable to those who enrol participants or assign interventions: N/A |
| Allocation concealment mechanism | 16b | Mechanism of implementing the allocation sequence (eg, central telephone; sequentially numbered, opaque, sealed envelopes), describing any steps to conceal the sequence until interventions are assigned: N/A |
| Implementation | 16c | Who will generate the allocation sequence, who will enrol participants, and who will assign participants to interventions: N/A |
| Blinding (masking) | 17a | Who will be blinded after assignment to interventions (eg, trial participants, care providers, outcome assessors, data analysts), and how: N/A |
|  | 17b | If blinded, circumstances under which unblinding is permissible, and procedure for revealing a participant’s allocated intervention during the trial: N/A |
| **Methods: Data collection, management, and analysis** | | |
| Data collection methods | 18a | Plans for assessment and collection of outcome, baseline, and other trial data, including any related processes to promote data quality (eg, duplicate measurements, training of assessors) and a description of study instruments (eg, questionnaires, laboratory tests) along with their reliability and validity, if known. Reference to where data collection forms can be found, if not in the protocol:  Assessments will be conducted at two timepoints, by the lead researcher, Fiona Kennedy, prior to commencement of the programme (Time 1: Feb-April 2024) and at programme completion (Time 2: May-June 2024). Fiona Kennedy is trained in the use of all study outcomes and is an experienced physiotherapist. The study instruments and outcomes measures are described in the protocol and summarised in Table 1 and 2. The test battery (secondary outcome measures) was developed by consensus with experts in the area of physical function, frailty and inclusion health and all measures were chosen based on their psychometric properties and practical utility. As frailty is closely associated with advancing age, these measures are mostly extrapolated from the geriatric setting and have proven validity and reliability. The data collection form is attached (Supplementary file: S3).  For the qualitative part, interview data will be collected using an interview schedule (Supplementary file: S2) |
|  | 18b | Plans to promote participant retention and complete follow-up, including list of any outcome data to be collected for participants who discontinue or deviate from intervention protocols:  All participants will be invited to complete the final assessment regardless of programme completion status. |
| Data management | 19 | Plans for data entry, coding, security, and storage, including any related processes to promote data quality (eg, double data entry; range checks for data values). Reference to where details of data management procedures can be found, if not in the protocol:  All data will be pseudonymised at point of entry into excel spreadsheets in Trinity College Dublin’s (TCD) provided and supported secure Sharepoint cloud-based system on the TCD initialised and encrypted laptop of the lead research physiotherapist and then transferred into IBM SPSS V28 for analysis. This system will be shared with the Principal Investigator only. |
| Statistical methods | 20a | Statistical methods for analysing primary and secondary outcomes. Reference to where other details of the statistical analysis plan can be found, if not in the protocol:  For quantitative data, nominal or ordinal variables will be reported as frequencies and percentages. Continuous variables will be summarised as mean and standard deviation if normally distributed and median and inter-quartile range if non-normally distributed. Data will be tested for normality using the Kolmogorov–Smirnov/Shapiro Wilk test and will be compared across timepoints using the general linear model procedure (normally distributed data) and the Friedman’s test (non-normally distributed data). Chi-squared t-tests will be used where appropriate and some data may be categorised to investigate relationships between variables. Exploratory regression models will be developed to explore correlates and predictors of frailty and poor physical functioning. A *p*-value of <0.05 will be considered significant.  For the qualitative data, Braun and Clarke thematic analysis methodology will be employed to provide an in-depth analysis of the exit interview data. |
|  | 20b | Methods for any additional analyses (eg, subgroup and adjusted analyses):  Retention analysis will be categorised to look for patterns of attendance (Protocol, Table 1).  Subgroup analysis will be conducted in younger and older participants and in pre and post-menopausal women. |
|  | 20c | Definition of analysis population relating to protocol non-adherence (eg, as randomised analysis), and any statistical methods to handle missing data (eg, multiple imputation):  Due to the feasibility focus of the study a complete case analysis will be undertaken. |
| **Methods: Monitoring** | | |
| Data monitoring | 21a | Composition of data monitoring committee (DMC); summary of its role and reporting structure; statement of whether it is independent from the sponsor and competing interests; and reference to where further details about its charter can be found, if not in the protocol. Alternatively, an explanation of why a DMC is not needed:  This study does not have a formal DMC. It is being undertaken by Fiona Kennedy as part of her PhD and is supervised by the Principal Investigator, Dr. Julie Broderick and co-supervisor Dr. Deirdre Murray. Prof Roman Romero-Ortuno and Prof Clíona Ní Cheallaigh are study collaborators and will collaborate and advise on the data interpretation and reporting. |
|  | 21b | Description of any interim analyses and stopping guidelines, including who will have access to these interim results and make the final decision to terminate the trial:  Interim analysis will take place on a monthly basis while the study is ongoing. Dr. Julie Broderick will have access to the study data and will make the final decision, if necessary, to terminate the trial. |
| Harms | 22 | Plans for collecting, assessing, reporting, and managing solicited and spontaneously reported adverse events and other unintended effects of trial interventions or trial conduct:  If an adverse event occurs a participant will be advised to seek medical attention and will followed up accordingly. If advised in writing by a medical practitioner, the participant may return to the programme. |
| Auditing | 23 | Frequency and procedures for auditing trial conduct, if any, and whether the process will be independent from investigators and the sponsor  N/A to this study |
| Ethics and dissemination | | |
| Research ethics approval | 24 | This research has been approved by the Faculty of Health Sciences, Trinity College Dublin, (Ethical Approval Reference Number: 211202). |
| Protocol amendments | 25 | Plans for communicating important protocol modifications (eg, changes to eligibility criteria, outcomes, analyses) to relevant parties (eg, investigators, REC/IRBs, trial participants, trial registries, journals, regulators):  Any important protocol modifications will be reported to all relevant parties. |
| Consent or assent | 26a | Who will obtain informed consent or assent from potential trial participants or authorised surrogates, and how (see Item 32):  Written informed consent will be obtained from trial participants by the lead researcher, Fiona Kennedy. |
|  | 26b | not applicable |
| Confidentiality | 27 | How personal information about potential and enrolled participants will be collected, shared, and maintained in order to protect confidentiality before, during, and after the trial:  Personal information will be collected from enrolled participants following consent using a data collection form (S2 Fig) and stored in a Trinity College Dublin provided and supported Sharepoint cloud-based system and all data will be stored in line with General Data Protection Regulation. Data will be pseudonymised at point of entry. |
| Declaration of interests | 28 | The authors have no competing interests to declare in connection with this article. |
| Access to data | 29 | Statement of who will have access to the final trial dataset, and disclosure of contractual agreements that limit such access for investigators:  The lead researcher Fiona Kennedy and her supervisor and the Principal Investigator will have access to the final dataset. |
| Ancillary and post-trial care | 30 | The risk of harm in this trial is low and no provisions are made for post-trial care, if any, for ancillary and post-trial care, and for compensation to those who suffer harm from trial participation |
| Dissemination policy | 31a | Plans for investigators and sponsor to communicate trial results to participants, healthcare professionals, the public, and other relevant groups (eg, via publication, reporting in results databases, or other data sharing arrangements), including any publication restrictions:  Following data analysis and write up results will be presented at relevant conferences and published in peer-reviewed journals. Results will also be presented to stakeholders in Merchants Quay Ireland. |
|  | 31b | Authorship eligibility guidelines and any intended use of professional writers  Listed study authors will have made a significant contribution. No professional writers will be involved. |
|  | 31c | Plans, if any, for granting public access to the full protocol, participant-level dataset, and statistical code:  Full access will be granted to the protocol. The data underlying this study cannot be shared publicly for the privacy of individuals that will participate in this study. The data will be shared on reasonable request to the corresponding author. |
| Appendices |  |  |
| Informed consent materials | 32 | Informed Consent Form and Participant Information Leaflet attached |
| Biological specimens | 33 | not applicable |

*It is strongly recommended that this checklist be read in conjunction with the SPIRIT 2013 Explanation & Elaboration for important clarification on the items. Amendments to the protocol should be tracked and dated. The SPIRIT checklist is copyrighted by the SPIRIT Group under the Creative Commons “[Attribution-NonCommercial-NoDerivs 3.0 Unported](http://www.creativecommons.org/licenses/by-nc-nd/3.0/)” license.

**PIL and ICF**

Participant Information Leaflet

| **Study Title** | **A FEASIBILITY STUDY TO EXPLORE THE ROLE OF A LOW THRESHOLD EXERCISE PROGRAMME IN A DAY CARE FACILITY FOR WOMEN WITH PROBLEMATIC DRUG USE AND HOMELESSNESS.** |
| --- | --- |
| **Research Site(s)** | Merchants Quay Ireland (MQI)-Riverbank, Dublin and Jane’s Place, Dublin. |
| **Principal Investigator(s) and**  **Co-Investigator(s) (Study Team)** | **Principal Investigator: Dr. Julie Broderick**  **Co-investigators:**   - **Fiona Kennedy, PhD student, Discipline of Physiotherapy, School of Medicine Trinity College** - **Prof. Cliona Ní Cheallaigh, Associate Professor and Consultant in Infectious Diseases, School of Medicine Trinity College** - **Prof. Roman Romero-Ortuno, Associate Professor and Consultant Physician, School of Medicine Trinity College** - **Dr. Deirdre Murray, Assistant Professor, Academic Unit of Neurology, Trinity College Dublin** |
| **Study Organiser/ Sponsor** | Trinity College Dublin/Irish Research Council |
| **Data Controller** | Trinity College Dublin |
| **Data Protection Officer** | Data Protection Officer  Secretary’s Office  Trinity College Dublin  Dublin 2 |

We would like to invite you to take part in a research study that is being carried out by Dr. Julie Broderick and her research team at Trinity College Dublin.

Before you decide whether or not you wish to take part, please take time to read this information leaflet carefully and discuss it with your family, friends or GP if you wish.

If there is anything which is not clear, or if you would like more information, please ask the researchers. You should understand the benefits and any risks of taking part in this study so that you can make a decision that is right for you.

| **Do I have to take part?** |
| --- |

No, you don’t have to take part in this study. It is entirely voluntary and up to you. If you decide not to take part, it won’t affect the current or future services you receive from Merchants Quay Ireland (MQI). Don’t feel rushed or under pressure to take part or to make a quick decision. You can change your mind and opt out even if the study has started.

This leaflet has six parts:

Part 1 - The Study

Part 2 - Data Protection

Part 3 - Approval, Organising and Funding

Part 4 - Future Research

Part 5 - Further Information

Part 6 – Next steps

Part 1 - The Study

| **Why have I been invited to take part?** |
| --- |

We are interested in understanding the role of exercise and nutrition on physical health in people experiencing homelessness and addiction. You have been invited to take part as you are accessing services in MQI (Jane’s Place or Riverbank). We are hoping to have 40-50 participants in the study.

| **Why is this study being done?** |
| --- |

We are doing this study to explore how well an exercise programme with a protein drink afterwards, works in MQI, Riverbank and Jane’s Place women’s only service for people experiencing problems with addiction and homelessness in Dublin 2 and 8.

| **What does taking part involve?** |
| --- |

This study is expected to commence in February 2024 and will continue for 3 months. The study will take place in MQI (Jane’s Place and Riverbank). If you decide to take part, a member of the research team will discuss this information leaflet and the consent form with you. You will be given a copy of your signed consent form and this leaflet to keep.

Following consent there are 3 parts to the study:

(1) You will complete an initial assessment, involving simple physical tests (outlined below) and a few questionnaires with the research physiotherapist, Fiona Kennedy. She will ask your age, medical and social history and read out a few short questionnaires, which include questions about your physical ability, any medical conditions that you have, your diet and if you are experiencing any pain. If further information, for the purposes of this study, is required, the research physiotherapist may clarify information with MQI staff. The assessment is outlined here:

- Handgrip: Your grip will be measured using an instrument called a dynamometer. See Figure 2.
- The 30-second Chair Stand Test: This a quick test of your leg strength. You will be asked to stand up and down as many times as possible for 30 seconds. See Figure 1.
- The Single Leg Stance: This is a simple test of your balance. You will be requested to balance on one leg, first the right, then the left.
- 10m Walk Test: You will be asked to walk a distance of 10m and you will be timed.
- The 2-minute Walk Test: You will be asked to walk up and down a 15m distance for 2 minutes (if able) and the distance covered will be recorded.
- Then we will measure the muscle bulk of your upper arm and calf. This test will involve the study physiotherapist measuring the width of your upper arm and calf. See Figure 4.
- Next your weight and height will be checked. See Figure 3.

Figure 1 Chair Stand and Walk Tests Figure 2 Grip Strength


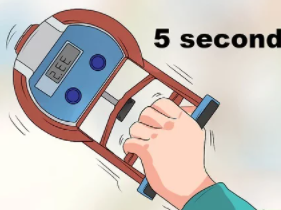

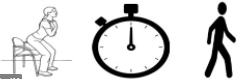


Figure 3 Weight and Height measurement Figure 4 Arm circumference


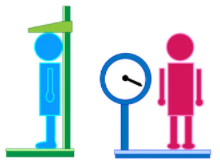

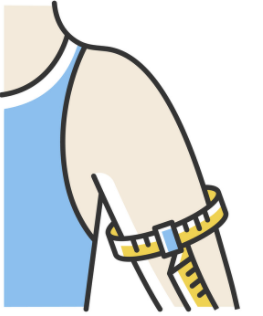


(2) The assessment will be followed by a 10-week exercise programme. The programme will involve two exercise classes per week and a Park Walk once per week. A protein drink will be offered to you after each exercise class.

(3) Following the exercise programme, you will be invited to do a one-to-one exit interview with the research physiotherapist Fiona, to get your feedback on the programme. This interview is entirely voluntary. During the interview you will be asked questions about your views on the programme, what you enjoyed about it and what were the challenges. We will arrange a time for your interview in MQI (Riverbank or Janes’ Place) with Fiona Kennedy. With your permission, the interviews will be audio recorded. We will transcribe the interview and offer you an opportunity to check and verify the transcript for up to 4 weeks following the invitation.

| **What are the possible benefits of taking part?** |
| --- |

You may see improvements in your mobility and physical health following the programme. In the future, findings from this study may help us design better exercise classes in settings similar to MQI (Riverbank and Jane’s Place). By participating, you are helping to advance science and medicine/education for future generations.

| **Are there any possible disadvantages or risks from taking part?** |
| --- |

- At all times, the well-being of participants takes priority over research activities.
- There is a very small chance of muscle soreness, an injury or fall as a result of the testing, but this risk is extremely low as the lead research physiotherapist will be closely supervising you and the physical tests are very brief.
- In the event of the interview triggering an emotional event, the interviewer will stop the session and we will link you in with an appropriate staff member in Riverbank or Jane’s Place.
- There is also a possibility that a connection to your identity could be made. However, we take many measures to ensure the confidentiality of all data and the risk to you of a breach of confidentiality is considered very low your data will be coded so this is unlikely and data from all participants will be analysed together, so the possibility of a connection to your identity is very unlikely.
- If you are harmed in any way, the researchers on this study are covered by insurance through Trinity College Dublin. This insurance will cover you in the unlikely event that you injured as a result of taking part in this study.

| **What will happen to the results of the study?** |
| --- |

The information in this study is being used in part fulfilment of the study physiotherapists PhD. The results of the study will be reported in medical/scientific/educational journals and disclosed at medical/scientific conferences. Some quotations from the interviews may be used in reports. However, no information which reveals your identity will be disclosed.

You will not be told the outcome of this study, but if you have questions about your own physical condition, the physiotherapist will be able to answer those questions.

Part 2 - Data Protection

| **What information about me (personal data) will be used for this study** |
| --- |

We will be using some personal information in our research to help us to identify problems with physical function. We will also use the information you provide in the audio recording, if you proceed with the exit interview, following the exercise programme.

We will be seeking your age, weight, height and information relating to any medical conditions you may have. As part of the assessment, we will be finding out about your physical condition, your nutritional status and any pain you may have.

Only the information which we need about you for this study will be collected and nothing extra. With your consent, we will inform your GP of your participation in this study using a standard letter which will be posted within 24 hours of your assessment. Once this letter has been posted your GP details will be deleted.

| **Who will access my personal data?** |
| --- |

Only the principal researcher, Dr. Julie Broderick and the study physiotherapist, Fiona Kennedy will be able to identify you. They will keep the master file which links your identity to the research data (your health data and your interview transcript).

The study physiotherapist Fiona Kennedy will replace your name with a code on all research data.

| **How is the information kept confidential and secure?** |
| --- |

Your privacy is important to us. We take many steps to make sure that we protect your confidentiality and keep your data safe. Here are some examples of how we do this:

- To protect your identity, we will replace your name with a code.
- The key to this code will be stored securely and separately from the other research information. .
- Any identifying information will be removed from the interview transcript.
- **Data security arrangements are** in place. Only TCD provided secure systems will be used.
- **A Data Protection Impact Assessment** has been carried out and the risk identified was low.
- No **presentation or publication** in relation to the study could identify you as all results will be analysed together.
- The research team having access to the personal data are **bound by a professional code of secrecy**.
- **Training in data protection law** and practice has been provided to the research team.

| **How long will my personal data be needed?** |
| --- |

The research data (data concerning health and physical function and the coded transcripts) will be retained until three years following the study physiotherapist’s PhD examination process is completed which is expected to be in November 2028. At that point, the link between you and your personal data will be securely deleted.

The audio recording of the interview will be retained until it has been transcribed and the content verified after which it will be securely deleted. This is expected to take place within 4 weeks of the interview. The transcript with identifiable information removed, will be retained until three years following the study physiotherapist’s PhD examination process is completed which is expected to be in November 2028.

Your consent form will be retained until the study physiotherapist’s PhD examination process is completed and will then securely deleted.

| **What is the lawful (legal) basis to use my personal data?** |
| --- |

We will only use your personal data for this research project, which we hope will improve the health of people experiencing homelessness and addiction. We will also ask for your consent as a requirement of Irish law (Health Research Regulations), but we do not rely on this as our legal basis under GDPR^1^.

The European General Data Protection Regulation (GDPR)

*Article 9(2) (j))*

*(Article 6(1)(e)*

| **What are my rights under Data Protection law?** |
| --- |

You are entitled to:

- The right to access to your data and receive a copy of it.
- The right to restrict or object to processing of your data.
- The right to object to any further processing of the information we hold about you.
- The right to have inaccurate information about you corrected or deleted.
- The right to request deletion of your data.

*By law you can exercise the following rights in relation to your personal data, unless the request would make it impossible or very difficult to conduct the research. You can exercise these rights by contacting your study researcher Fiona Kennedy or the Trinity College Data Protection Officer, Secretary’s Office, Trinity College Dublin, Dublin 2, Ireland. Email:* [*dataprotection@tcd.ie*](mailto:dataprotection@tcd.ie)*. Website:* [*www.tcd.ie/privacy*](http://www.tcd.ie/privacy)*.*

Part 3 - Approval, Organising and Funding

| **Has this study been approved by a research ethics committee?** |
| --- |

Yes, this study has been approved by the Faculty of Health Sciences Research Committee in Trinity College Dublin. Approval was granted on [INSERT DATE].

| **Who is organising and funding this study?** |
| --- |

This study is being undertaken by Fiona Kennedy as part of her Ph.D in Trinity College Dublin. This study is being funded by The Irish Research Council (IRC) and Trinity College Dublin. The IRC will be provided with an anonymous report. They will not access any personal data.

| **Will I be paid for taking part?** |
| --- |

No, there is no payment for taking part in the study.

| **Will my data be used for future studies?** |
| --- |

Basic demographic details provided for the purposes of this study, such as age, biological sex, living arrangements and addiction status may be collectively compared to future studies.

Part 4 - Further Information

| **What happens if I change my mind?** |
| --- |

Your participation in this study is voluntary and you can change your mind even if the study has started.

You do not have to give a reason for changing your mind.

If you would like to withdraw from the study, please contact Dr. Julie Broderick at (01) 8962110, who can organise this for you. She will discuss with you if you are happy for us to continue to use information about you (personal data) which has already been collected. If you do not consent to your personal data being retained for this study, we will delete any information that could identify you.

Please note that we will not be able to remove personal data which has been shared or pooled for use in publication before your request for deletion.

We will not contact you again.

| **Who should I contact for information or concerns?** |
| --- |

If you have any concerns or questions, you can contact:

- Principal Investigator: Dr. Julie Broderick (01) 8962110

If you have any questions in relation to your rights under data protection law, you can contact the Data Protection Officer, Trinity College Dublin: Data Protection Officer, Secretary’s Office, Trinity College Dublin, Dublin 2, Ireland. Email: dataprotection@tcd.ie. Website: [www.dataprotection.ie](http://www.dataprotection.ie/) .

Under GDPR, if you are not satisfied with how your data is being processed, you have the right to raise a concern with the Office of the Data Protection Commission, 21 Fitzwilliam Square South, Dublin 2, Ireland. Website: [www.dataprotection.ie](http://www.dataprotection.ie/)

Part 5 - Next Steps

| **Will I be contacted again?** |
| --- |

If you would like to take part in this study, you will be asked to sign the Consent Form on the next page. You will be given a copy of this Leaflet and the signed Consent Form to keep. Please retain these in case they are needed for future reference.

After the study you will not be contacted again by the study team.

**Thanks**

Thank you for taking the time to read this Participant Information Leaflet.

**Informed Consent Form**

| **STUDY: A FEASIBILITY STUDY TO EXPLORE THE ROLE OF A LOW THRESHOLD EXERCISE PROGRAMME IN A DAY CARE FACILITY FOR WOMEN WITH PROBLEMATIC DRUG USE AND HOMELESSNESS.**  **Recruitment Site: Merchants Quay Ireland-Riverbank and Jane’s Place** | |
| --- | --- |
| There are **two sections** in this form.  **Section 1** contains statements of understanding and asks you to tick each if you understand. Please ask any questions you may have when reading each of the statements.  **Section 2** asks for your informed consent. Please select either ‘yes’ or ‘no’ to indicate your choice.  Thank you for participating.  The end of this form is for the researchers to complete. | |
| **1. General Understanding** | **Tick** |
| I confirm that I have read and understood the Information Leaflet for the above study. The information has been fully explained to me and I have been able to ask questions, all of which have been answered to my satisfaction. |  |
| I understand that taking part in this study is entirely voluntary. I understand that not taking part will have no negative impact on me. |  |
| I understand that I can leave this study at any time without giving a reason. I understand that leaving this study will not affect the services I receive from MQI, now or in the future. |  |
| I understand that all information about me will be kept private and confidential and that my name will not be disclosed in any publication. |  |
| I understand that I will not be paid for taking part in this study. |  |
| I know how to contact the research team if I need to (contact details below). |  |
| **By ticking each box above and choosing my options below and signing this document I agree to participate in ‘X’ study as described in the Participant Information Leaflet.** | |
| **2. Consent** |  |
| I agree to take part in this research study, having been fully informed of the risks and benefits in the participant information leaflet provided to me. | ***Yes No***   |  |  | | --- | --- | |
| I agree to the use of information about me (personal data) including information from the exit interviews being used by the research team for this research study as described in the participant information leaflet. | ***Yes No***   |  |  | | --- | --- | |
| I consent to the research team informing my GP about my participation in this study. | ***Yes No***   |  |  | | --- | --- | |
| I consent to the research team clarifying information with MQI staff for my safety, where necessary for the purposes of this research study only. | ***Yes No***   |  |  | | --- | --- | |

______________________________________________________________________________________

Participant Name (Block Capitals) Participant Signature Date

______________________________________________________________________________________

Witness Name (Block Capitals) Witness Signature Date

**To be completed by the Principal Investigator or nominee.**

I, the undersigned, have taken the time to fully explain to the above participant the nature and purpose of this study in a way that they could understand.

I have explained the risks and possible benefits involved. I have invited them to ask questions on any aspect of the study that concerned them.

I have given a copy of the participant information leaflet and consent form to the participant with contact details of the study team.

Researcher name _________________________________________________

Title and qualifications _________________________________________________

Contact details: Fiona Kennedy, Discipline of Physiotherapy, Trinity Centre for Health Sciences, St. James’s Hospital, Dublin 8, (phone : 01-8962110)

Signature _________________________________________________

Date _________________________________________________

**Copies to be created and retained: 1 for Participant, 1 for PI**
