# Supplementary material for: Evaluation of a Low-threshold Exercise And Protein supplementation intervention for Women (LEAP-W) experiencing homelessness and addiction: Protocol for a single-arm mixed methods feasibility study
Source: PLoS One. 2025 Feb 6;20(2):e0300412. doi: 10.1371/journal.pone.0300412 (PMC11801605; doi:10.1371/journal.pone.0300412)
Supplement: S4 File — (DOCX) [file pone.0300412.s004.docx]

**S4: Primary and Secondary Outcomes**

| **Variable** | **Test/Measurement Scale** | **Definition** | **Unit of measurement** | **Measurement Category** | **Statistical method** | **Statistical technique** |
| --- | --- | --- | --- | --- | --- | --- |
| Recruitment | Record of number recruited (count) | Number of eligible consenting participants who completed the initial assessment | Number | Primary Outcome | Descriptive | Frequencies/percentages |
| Retention | Record of number recruited (count) | Number of return visits and the frequency of attendance  -*attending at least once/week for*  *> 50% of the duration of the programme (regular attenders)*  *-attending at least once/week for*  *< 50% of the duration of the programme (sporadic attenders)*  *-did not return (non-attenders)* | Number | Primary Outcome | Descriptive | Frequencies/percentages |
| Adherence | Record of number recruited (count) | Adherence to the exercise and protein supplement | Number expressed as percentage | Primary Outcome | Descriptive | Frequencies/percentages |
| Physical function | 10m Walk Test  (continuous) | Gait speed measured over a 10m walkway. | Distance (m/s) | Secondary Outcome | Descriptive/Inferential | a or b* |
|  | 2-Minute Walk Test  (continuous) | Functional mobility measured in m over a 15m walkway. | Distance (m) | Secondary Outcome | Descriptive/Inferential | a or b* |
| Lower limb strength | Chair Stand Test  (count) | Number of full sit-to-stand repetitions performed in 30s. | Number of stands | Secondary Outcome | Descriptive/Inferential | a or b* |
| Upper limb strength | Grip strength  (continuous) | Hand grip dynamometry. | Dynamometer score (kg) | Secondary Outcome | Descriptive/Inferential | a or b* |
| Balance | Single Leg Stance Test  (continuous) | Timed unassisted standing on 1 leg, without the free leg touching standing leg, eyes open & hands placed on the hips. | Seconds | Secondary Outcome | Descriptive/Inferential | a or b* |
| Limb circumference | Upper and lower limb circumference  (continuous) | Mid-arm and mid-calf circumference, measured in cms using tape measure. | Width (cm) | Secondary Outcome | Descriptive | Frequencies/percentages |
| Pain | Numerical Pain Rating Scale  (ordinal) | Uni-dimensional measure of pain intensity. | 0-10 (0 =no pain, 10=worst pain) | Secondary Outcome Measure | Descriptive/Inferential | a or b* |
| Frailty | Clinical Frailty Scale  (ordinal) | Measures multi-dimensional frailty. Judgement-based, 9-point scale. | 1-9 (1=very fit, 9=terminally ill) | Secondary Outcome Measure | Descriptive/Inferential | -a or b*  -Frequencies/percentages for frailty categories  -Linear regression and logistic regression tests |
|  | SHARE-Frailty Instrument (ordinal and continuous) | Measures physical frailty. 4 brief questions relating to (i) exhaustion, (ii) loss of appetite, (iii) walking difficulties & (iv) low physical activity & (v) one objective measure of grip strength. | Frailty category (non-frail, pre-frail or frail) and frailty numerical score | Secondary Outcome Measure | Descriptive/Inferential | -a or b*  -Frequencies/percentages for frailty categories  -Linear regression and logistic regression tests |
| Nutritional Status | Mini-Nutritional Status  (ordinal) | Assesses the risk of malnutrition, six questions relating to (i) food intake, (ii) weight loss, (iii) mobility, (iv) psychological stress, or acute disease, (v) the presence of dementia or depression, and (vi) body mass index (BMI) | 0-14 scale:  0-7 = malnourished  8-11 = at risk of malnutrition  12-14 = normal nutritional status | Secondary Outcome Measure | Descriptive/Inferential | a or b*  Frequencies/percentages for frailty categories |
| Overall health status | Short Form-12  (continuous) | 12-question, self-report measurement of health status encompassing physical and social activities, pain, mental health, emotional health, vitality, and general health perceptions | Results entered to a software programme to provide 2 scores; Physical Component Score and Mental Component Score. | Secondary Outcome Measure | Descriptive/Inferential | a or b* |

*a= mean/SD & paired t-tests used if data is normally distributed, b= median/IQR & Wilcoxin signed-rank test used if data is not normally distributed
