# Supplementary material for: Evaluation of a Low-threshold Exercise And Protein supplementation intervention for Women (LEAP-W) experiencing homelessness and addiction: Protocol for a single-arm mixed methods feasibility study
Source: PLoS One. 2025 Feb 6;20(2):e0300412. doi: 10.1371/journal.pone.0300412 (PMC11801605; doi:10.1371/journal.pone.0300412)
Supplement: S5 File — (DOCX) [file pone.0300412.s005.docx]

**Protocol approved by Trinity College Dublin, Faculty of Health Sciences Research Ethics Committee on 18.01.2024. Ref: 211202**

**Title:**

Evaluation of a Low threshold Exercise And Protein supplementation intervention for Women (LEAP-W) experiencing homelessness and addiction. Protocol for a single-arm mixed methods feasibility study.

**Dates and Duration of Study**:

February 2024 to July 2024.

**Primary Location for data collection**:

Merchants Quay Ireland Women’s Service (Jane’s Place).

**Research aims and objectives**:

The overall aim of this project is to develop preliminary data which tests the feasibility of a fitness focused drop-in exercise programme with dietary supplementation to target physical functioning in women who are homeless and experiencing addiction.

**Study Objectives:**

1. To evaluate the adherence (uptake, compliance, number of repeat visits) to a drop-in exercise and nutritional supplementation programme.
2. To explore the physical and nutritional status of participants.
3. To explore pain in participants.
4. To establish the difference in physical/nutritional status of participants.
5. To ascertain perception of unmet physical health needs, exercise habits and how an exercise intervention should ideally be designed to meet the needs of this cohort with lived experience of homelessness and active addiction issues.
6. To understand barriers and facilitators of engaging in physical activity interventions.

**Background:**

The area of Inclusion health is a ‘newer’ area of focus in medicine. This considers the health of vulnerable people such as those who are homeless. Lately the very poor health status of people who are homeless is beginning to receive attention. People who are homeless are more likely to experience poor mental health and addiction as well as suffering from non-communicable diseases. Our previous research has provided concrete evidence of frailty and accelerated physical ageing among people experiencing homelessness (Kiernan et al, 2021). Appropriate exercise/physical rehabilitation strategies can stabilise or reverse frailty and general physical decline, but it is not known how an intervention would work in practice in this population. As the concept of frailty can be problematic in people under the age of 65, this intervention will focus on the opposite of frailty which is fitness.

This study based will take place in Merchants Quay (Riverbank Centre and Jane’s Place) from Feb-July 2024, funded by an Irish Research Council New Foundations Grant. The study collaborators are Prof. Cliona Ní Cheallaigh who is an expert in Inclusion Health and the health of vulnerable populations. She is also a member of the Board of Merchants Quay Ireland. Prof. Roman Romero-Ortuno is an expert in the area of frailty and will advise on this aspect of the programme. Prof Ní Cheallaigh and Prof Romero-Ortuno will collaborate on an advisory capacity and will collaborate in the write up of this study for publication. Dr. Deirdre Murray has joined the study team in an advisory capacity. Fiona Kennedy (FK), a Physiotherapist with over 20 years clinical experience and a PhD student funded through the 1252 award will lead the delivery of the programme She was employed as a Research Assistant, delivering a previous programme in Merchants Quay. A Research Assistant will also be employed on this study.

**Research Approach:**

The intervention will be a three-times weekly (for 10-weeks), multi-modal exercise programme with dietary supplementation. The approach will be low threshold, meaning there will be flexibility in programme delivery. The exercise programme will be mainly focus on all round fitness including resistance aerobic and functional exercises, with in-built flexibility based on individual participants’ needs. To promote post exercise muscle protein synthesis, a nutritional supplement (200ml pre-prepared ‘protein shake’ Fresubin) which consists of 20g of protein will be offered to all participants who will drink this in the exercise room straight after exercise. In an attempt to build sustainability beyond the life cycle of the project, participants will also be educated about exercise and available local resources and will receive information about protein supplementation through their diet rather than nutritional supplementation. In a further effort to build sustainability into the programme, it will also include an optional ‘park walk’ – a 30 minute self-paced, low-intensity social ‘walk and talk’. The physiotherapy researcher will deliver the intervention. The exercise classes will take place on Mondays and Wednesdays and the Park Walk will take place on Friday mornings.

To get programme feedback and an in-depth understanding of the perspectives of the participants, following the exercise programme all participants will be invited to take part in a one-to-one exit interview, which will be audio-recorded and transcribed by the study researcher Fiona Kennedy. The interviews will take part in a private room in Merchants Quay (Jane’s Place or Riverbank). We expect that some participants will attend just once due to the transient nature of this cohort but repeat visits will be encouraged.

**Design**:

Single arm feasibility mixed methods study.

**Data Collection methods:**

Data will be pseudo-anonymised and entered in Merchants Quay in a Trinity College Dublin double encrypted laptop at the point of data collection.

**Sample size:**

Due to the novelty of this area, there is no data on which to base a power analysis. Based on available audit data from consultation with Merchants Quay Ireland, we plan to recruit at least 24 participants.

**Recruitment:**

The appointed gatekeeper will be the main study liaison. While the Participant Information Leaflet (PIL) and consent form about the programme will be distributed by the gatekeeper, there may be multiple pathways to the programme from staff members who will be aware which clients may engage with the service and may suggest to them that they seek information about the programme. Leaflets in plain English will be available throughout Merchants Quay (Riverbank (mixed service) /Jane’s Place (women’s only service).

**Inclusion criteria:**

The aim of the study is to be as pragmatic and low threshold as possible, therefore any person, identifying as a woman, accessing services in Merchants Quay will be eligible to take part, they can be at any point of the addiction journey.

**Exclusion criteria:**

- People identifying as male.
- Potential participants would be excluded if there was a major reason such as behavioural, acute confusion, agitated state or major physical problems (medical or orthopaedic) which would preclude ability to participate safely in exercise class.
- Confirmed pregnancy (tests outlined in this study are not deleterious to people who are pregnant but as people with an advanced pregnancy may score differently on physical functioning/performance tests, people with a confirmed pregnancy will be excluded from study participation to limit bias).

**Consent:**

Written informed consent is a prerequisite to taking part in this study. Interested participants will receive the PIL, outlining the details of the research study, and the consent form. Bearing in mind the expected high levels of functional illiteracy among participants, the study and consent form will be explained verbally in lay language as appropriate. Time will be given to ensure all queries will be answered. If potential participants are happy to proceed, the consent form will be signed and dated. The research physiotherapists will also sign the consent form.

We are however seeking some flexibility in terms of the seven-day gap between provision of the PIL and study participation. This is due to the transient nature of this population and likely fluctuation in interest levels due to other competing priorities related to active addiction issues and issues related to mood and motivation. We have therefore been advised by senior staff in Merchants Quay (Riverbank/Jane’s Place) that if clients are willing and engaged at that time to participate that they should be able to participate with a flexible gap between getting the information about the study and participation. This method was employed previously in a cross-sectional study we conducted with patients in St. James’s Hospital and worked well in practice.

We also feel this is justified as (i) the test battery outlined in this protocol is just an expansion a standard physiotherapy assessment, (ii) the low intensity level of the exercise programme. Its innocuous nature means there is very little chance of any associated adverse effects so in some cases, consent will be gained after informing the potential participant about the study and data collection will then proceed if the patient is agreeable to participate and consent is gained.

If participants are agreeable to take part the consent form will be signed. The research physiotherapist will continue to check after each test and during the exercise programme that the patient is still happy to continue, and breaks will be provided, or the session will be terminated where necessary.

**Data subject rights:**

- Right of access. Participants will have the right to access their data until the point at which it is published, as after that point individual data cannot be removed.
- Right to rectification. Participants will have the right to rectify their data until which point that data is published. After that, individual data cannot be removed.
- Right to erasure. Participants will have the right for their data to be erased until the point at which it is published, as after that point individual data cannot be removed.
- Right to object to processing based on legitimate or public interest. Participants have the right to object to processing their data based on legitimate or public interest.
- Right to data portability. If participants wish they have the right to obtain and reuse their personal data for their own purposes across different services.
- Right to object to profiling or making decisions about individuals by automated means. This will not apply to this study as there will be no profiling or making decisions by automated means. If participants wish to withdraw from the study, they can contact the Principal Investigator listed in the PIL.

**Ethical issues:**

This feasibility study includes some physical tests which are an expanded physiotherapy assessment. The intervention is a safe volume of low–moderate intensity exercise in a supervised setting. No ethical issues are anticipated in this study.

**Potential for adverse outcome:**

Generally, the benefits of exercise far outweigh any risks but there is a noted higher incidence of injury in certain sports such as soccer, team sports, skiing, tennis, running or athletics. There is no increased risk of injury with routine physical activity such as walking or gardening (Pons-Villanueva et al, 2010).

As the exercise in this programme will mimic daily activities such as performing sit-to-stand and walking around cones as well there is very low potential for an adverse outcome other than slight muscle soreness, or rarely the risk of a low-level injury that may result from a bout of exercise. While this population is vulnerable, physically they are mobile and accessing services day services in Merchants Quay Ireland, therefore they are not expected to be majorly physically debilitated.

Every effort will be made to mitigate against this risk of an adverse outcome, such as:

(i) The PAR-Q pre-screening questionnaire will be conducted on participants prior to participation

An additional safety measure has been built into the intervention. If ‘yes’ is ticked to any of the PAR-Q screening questionnaire, the research physiotherapist will, with permission contact the relevant GP and ensure it is safe for the participant to participate in the exercise intervention. If the individual does not have a GP, FK will discuss this individual case with Prof. Clíona Ní Cheallaigh, a specialist in Inclusion Health based in St. James’s Hospital.

(ii) Exercise will be fully supervised by the research physiotherapist and personalised to each individual client and delivered by a trained physiotherapist with expertise in adapting exercise to suit the needs of people with complex physical and mental health needs.

(iii) Patients with significant mobility problems will be excluded from study participation so limiting the chance of a fall happening while exercising.

**Discussion of topics of a sensitive nature:**

As this intervention focusses on physical aspects rather than exploring personal topics, it is unlikely to be upsetting for participants. There is a small possibility that conducting physical exercises may remind clients of their poor or deteriorated physical status and be a source of embarrassment or upset. This is unlikely to happen though as the research physiotherapist will taper exercises to suit the individual ability of each participant.

In the case that participants do become upset, the research physiotherapist will be able to judge the situation and link the participant in with the medical/nursing staff/psychosocial support/services in MQI.

**Do participants undergo a clinical assessment: yes.**

**Secondary outcomes using a test battery will measure impact and include:**

- BMI (weight/height)
- Grip strength (hand grip dynamometry)
- 10-meter walk test
- 2-minute walk test
- 30-second chair stand test
- Single leg stance
- Bodily pain (yes/no, if yes – visual analogue scale to quantify)
- Frailty [SHARE frailty instrument and Clinical Frailty Scale]
- Nutritional Status [Mini-nutritional assessment]
- Quality of life - Short form 12
- An exit interview will be conducted, recorded and transcribed verbatim to ascertain perceptions about the programme and suggestions for the design of future programmes.

**Will participants GP be informed?**

Participants will be asked the name of their GP. If the GP in Merchants Quay is not specified as their GP, a letter indicating that the participant is taking part in this study will be sent to their own GP. If the GP in Merchants Quay is listed as their main GP, they will be informed that participants are taking part in this intervention.

**Permission to disclose information to GP:**

In the unlikely event that there is an injury or other adverse effect as a result of the exercise intervention, the participants GP will be informed. This detail is included in the PIL.

**Potential for benefit:**

(i) Small immediate benefit

Without wishing to overstate the benefits, with exercise there is an immediacy in impact as every bout of exercise is health enhancing, therefore the participants are likely to benefit in some way from this exercise intervention, especially if they attend regularly. There may also be an energy boost from the nutritional supplement. It is also possible that having a conversation around exercise will help participants chose to participate in this healthy lifestyle behaviour in the future.

(ii) Longer term benefit

It is unlikely that clients will benefit from medium to longer term benefits. Data generated may be used to inform a future full scale randomised controlled trial of exercise in this setting which may benefit this client group in the future.

**Funding:**

This study has been funded by a New Foundations Grant from the Irish Research Council. This will pay the salary of the Research Assistant appointed to work on this study. PhD student Fiona Kennedy is funded by the 1252 TCD research scholarship.

**Payment:**

No payment will be made to research participants.

**Ethical approval from another organisation:**

No, as there is no internal research ethics committee in Merchants Quay Ireland.

**Permission sough from another site outside of TCD to conduct this research:**

Yes, permission has been sought from Merchants Quay Ireland to conduct this study.
